# The Effect of Three Kinds of Surface Treatment Methods on the Corrosion and Wear Resistance of AM60B Magnesium Alloy with La, Ce Addition

**DOI:** 10.3390/ma19173711

**Published:** 2026-08-31

**Authors:** Shusen Wang, Zhongyu Qiu, Naibao Huang, Chenghao Liang, Wenning Jiang

**Affiliations:** 1 College of Public Security Management, LiaoNing Police College, Dalian 116036, China; 2 Materials Sciences & Engineering, Dalian Maritime University, Dalian 116026, China

**Keywords:** magnesium alloy, lanthanum cerium rare earth element, chemical conversion film, corrosion, friction and wear

## Abstract

Three kinds of surface treatments, including permanganate, molybdate, and phytic acid conversion films, were fabricated on La-Ce mischmetal containing AM60B magnesium alloy. Their effects on the corrosion and tribological behaviors of the alloy in 3.5 wt% NaCl solution were systematically investigated via Mott–Schottky analysis, electrochemical measurements, and friction–wear tests. The results show three surface treatments shift the flat band potential in the negative direction, reduce the corrosion current density, enlarge the electrochemical impedance arc radius, and decrease the friction coefficient, conferring remarkably enhanced corrosion and wear resistance to the alloy substrate. The performance enhancement is ascribed to the formation of uniform, dense conversion films that act as effective physical barriers, which impede the penetration of corrosive species, isolate the substrate from the aggressive aqueous environment, and improve the chemical and electrochemical stability of the alloy–solution interface. The comprehensive performance ranking of the three surface treatments in terms of corrosion and wear resistance is as follows: permanganate conversion film > molybdate conversion film > phytic acid conversion film.

## 1. Introduction

Magnesium alloys have gained extensive applications in automobiles, electronics, computers, transportation, telecommunication, and aerospace, benefiting from their low density, high specific strength, excellent fatigue limit, good casting properties, and favorable thermal conductivity. Nevertheless, the intrinsically high chemical reactivity of magnesium leads to poor corrosion resistance, which significantly hinders the full utilization of their performance advantages in practical service [1,2,3]. Chemical conversion treatment technology is an important means of corrosion protection on the surface of magnesium alloys because it has a reduced film-forming process, various conversion schemes, a low price, and can significantly improve the corrosion resistance of the magnesium alloy, which accounts for a large proportion of the surface treatment of the magnesium alloy. The traditional chemical conversion treatment technology is chromate treatment, a traditional form of chemical conversion [4] with a mature process, good adhesion, strong corrosion resistance, and inherent self-repair ability. However, the high toxicity of hexavalent chromium has led to increasingly stringent regulatory restrictions worldwide, making the development of chromium-free, eco-friendly conversion technologies a major research focus.

In recent years, a variety of chromium-free conversion systems have been explored for magnesium alloys, including phosphate [5], permanganate [6], phosphate–permanganic acid [7], molybdate [8], stannate [9], phytic acid [10], and rare earth salt conversion films [11]. Phosphate conversion films are cost-effective with good adhesion but exhibit porosity and moderate corrosion resistance [5]. Permanganate conversion films achieve corrosion protection comparable to chromate films with rapid film formation [6]. Phosphate–permanganic acid conversion films enhance film-forming capability but show non-uniform coverage on Al-containing magnesium alloys due to a preferential reaction in β-Mg_17_Al_12_ phases [7]. Molybdate conversion films form uniform, compact films with low toxicity, though microcracks are frequently detected in as-deposited layers [8]. Stannate conversion films serve well as pre-treatments for subsequent electroplating but require elevated processing temperatures and offer limited protection when used alone [9]. Phytic acid conversion films are distinguished by their non-toxicity, biocompatibility, and excellent adhesion to organic topcoats, although their thinness constrains independent barrier performance [10]. The highly complex RE conversion coatings also raise growing concerns regarding potential biological risks from abundant secondary phases [11].

AM60B magnesium alloy is a widely employed cast magnesium alloy in industrial applications. Relevant studies have demonstrated that adding lanthanum and cerium mixed rare earth can effectively enhance the corrosion resistance and wear resistance of AM60B magnesium alloy [12]. At present, the corrosion behavior of the magnesium alloy chemical conversion treatment process and the matrix of a magnesium alloy with a rare earth element are studied more [13,14], but the corrosion resistance of the La, Ce mixed rare earth AM60B magnesium alloy after chemical conversion treatment is less reported [15]. Numerous scholars have investigated the fabrication of micro-arc oxidation coating and nanocomposite coatings on the surface of a magnesium alloy [16,17]. Nevertheless, the approach of constructing a conversion film on the alloy surface via solution immersion, to simultaneously enhance the electrochemical and mechanical properties of the alloy, still exhibits promising development potential [18].

In this work, permanganate, molybdate, and phytic acid conversion films were selected for their complementary merits of fast formation of conductive oxide barriers, uniform low-toxicity passivation, and eco-friendly chelation with superior topcoat adhesion, respectively. The main purpose is to screen feasible surface treatments that simultaneously improve the corrosion and wear resistance of magnesium alloys, through comparative evaluation of the three surface treatments on La, Ce mixed rare earth AM60B.

## 2. Experimental Section

### 2.1. Materials

The experimental materials are AM60B magnesium alloy (AM60B-MM) with La and Ce rare earth elements. The chemical composition of AM60B is Al 5.97, Zn 0.14, Mn 0.31, Fe 0.002, Be 0.00082, Si 0.028, Cu 0.0033, Ni 0.00087, and the balance of Mg. That of AM60-MM is Al 5.48, Zn 0.13, Mn 0.30, Be 0.00082, Si 0.028, Cu 0.0033, Ni 0.00087, La 0.32, Ce 0.64 (mass fraction %), and the balance of Mg.

The sample sizes of wear and electrochemical tests were 20 × 30 × 1 mm and 10 × 10 × 1 mm, respectively. The sample was polished with water grinding sandpaper from 600# to 2000#, cleaned ultrasonically in deionized water and anhydrous ethanol, and then put in the desiccator for later use. The non-working face part was coated with insulating adhesive. The exposed work areas of the specimen for wear and corrosion tests were 20 × 30 mm and 10 × 10 mm, respectively. Each experiment was repeated at least three times, and the average value was used as the final result.

### 2.2. Preparation of Chemical Conversion Film for Magnesium Alloy

The chemical conversion film of magnesium alloy was prepared using three kinds of surface treatment methods, permanganate, acid salt and phytic acid. To obtain the complex film, the ratio of the sample area to the volume of the solution is above 1:100.

(1) Permanganate conversion membrane [19]

The composition of the conversion treatment solution was 150 g/L Na_2_HPO_4_, 50 mL/L H_3_PO_4_, 25 g/L KMnO_4_; temperature 80 °C, reaction time 30 min; repeated treatment 5 times. The transformed sample was immersed in Na_2_SiO_3_ 40 g/L solution, sealed at 90 °C for 30 min, washed with deionized water and anhydrous ethanol, and dried at room temperature.

(2) Molybdate conversion film [20]

The composition of the conversion solution was as follows: Na_2_MoO_4_ 10~20 g/L, NaF 2~4 g/L; the pH value of the solution was adjusted to 3.0 × 4.0 with H_3_PO_4_; the temperature was 60 °C. After being immersed in the solution for 30 min, the sample was taken out and washed with deionized water and anhydrous ethanol and dried at room temperature.

(3) Phytic acid conversion film [21]

Composition of the conversion treatment liquid was as follows: the ratio of ethanol to water was 9:1 (volume ratio), and the concentration of phytic acid was 0.6 mg/mL. The sample was soaked in alkaline solution (NaOH) of pH = 11 for 20 min, then treated in phytic acid–ethanol–aqueous solution for 30 min, then taken out and treated at constant temperature in heater at 130 °C for 30 min.

### 2.3. Experiment Method

The electrochemical performance of the magnesium alloy samples was tested with the Chenghua 660D electrochemical workstation. The three-electrode system was used as the working electrode, the saturated calomel electrode (SCE) was used as the reference electrode, and the platinum sheet was the auxiliary electrode. The test was carried out in an aqueous NaCl solution with a mass fraction of 3.5% at 25 °C. All potentials were relative to the saturated calomel electrode. After the sample was placed in the medium for 20 min, the AC impedance measurement and polarization curve were consecutively recorded in the same solution.

The scanning rate of the polarization curve test of the electrochemical sample was 60 mV/min, the frequency range of the AC impedance measurement was 10 m Hz to 100 kHz, the disturbance signal was 10 mV, and the measurement result was analyzed using ZSimp Win 3.6 software. The Mott–Schottky curve was recorded at 1 kHz with 5 mV sinusoidal excitation signal in the potential scan range −2.5 V to 0 V.

The friction coefficient was measured in 3.5%NaCl solution on the TE66 micro-abrasive wear tester (produced by Phoenix Tribology Laboratory, Berkshire, UK) after the sample was immersed in the solution for 20 min. The friction pair was made of Si_3_N_4_ ceramic ball (SCF1200, Φ = ~4 μm). During the process of wear, the load was fixed at 10 mN, the sliding distance was 10 mm, and the sliding speed was 10 mm/s. The total wear time was 10 min.

The morphology, element and phase analysis of the bare and treatment of magnesium alloy were observed via GSM-5600L scanning electron microscope (SEM, ThermoFisher, Shanghai, China) and D/MAX-Ultima + X-ray diffraction (XRD, Rigaku, Akishima, Tokyo, Japan). The new sample and solution were used for Mott–Schottky and friction coefficient tests, respectively.

## 3. Results and Discussions

### 3.1. Morphology and Composition of Three Chemical Conversion Films

**Figure 1:**
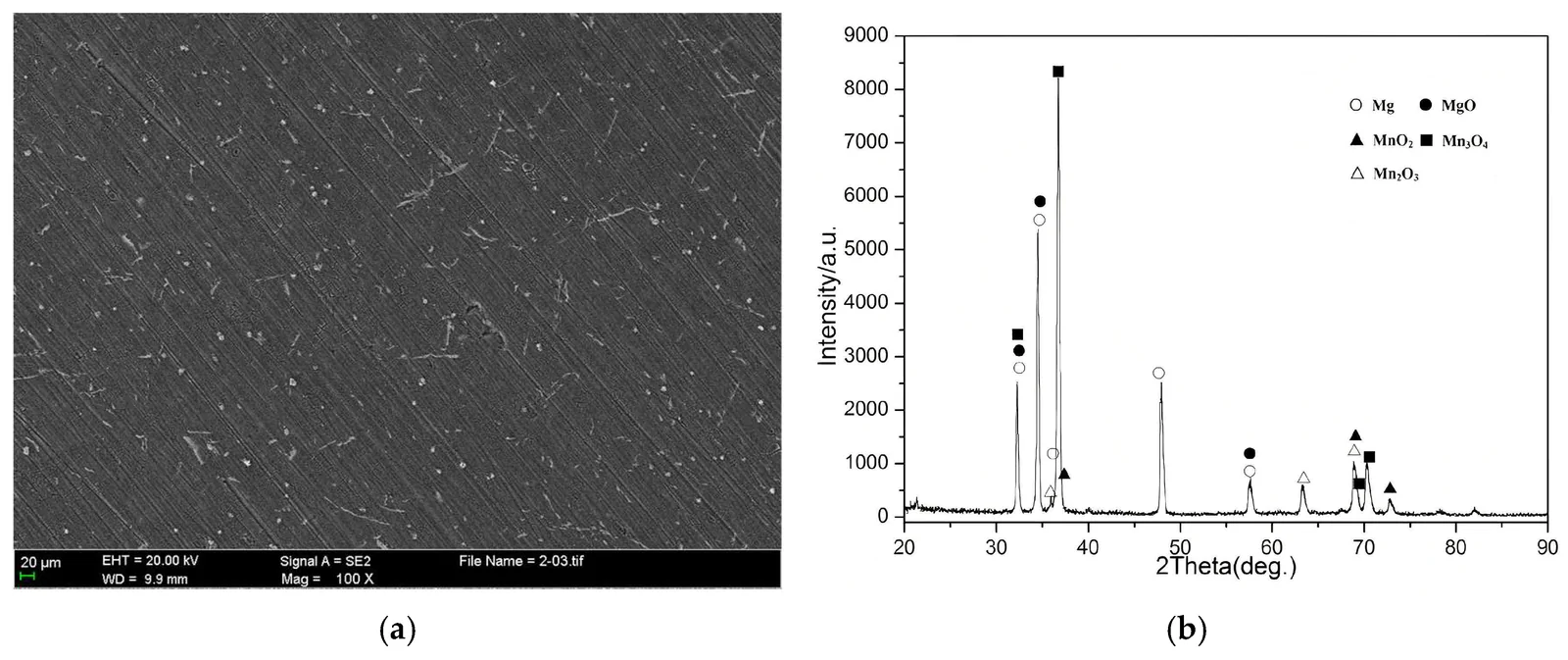
(**a**) Scanning electron microscope, and (**b**) X-ray diffraction of permanganate conversion film of AM60B-MM magnesium alloy.

Figure 1 presents the surface morphology and phase constitution of the permanganate conversion film fabricated on the AM60B-MM magnesium alloy, in which Figure 1a is a scanning electron microscopy (SEM) micrograph and Figure 1b shows the corresponding X-ray diffraction (XRD) pattern. SEM observations reveal that the permanganate conversion film possesses a uniform, dense and relatively smooth surface. Microcracks are visible on the coating surface, yet no critical defects such as pitting or peeling are observed. The XRD result (Figure 1b) confirms that the conversion coating is mainly composed of MgO and multiple manganese oxides, including MnO_2_, Mn_2_O_3_, Mn_3_O_4_.

**Figure 2:**
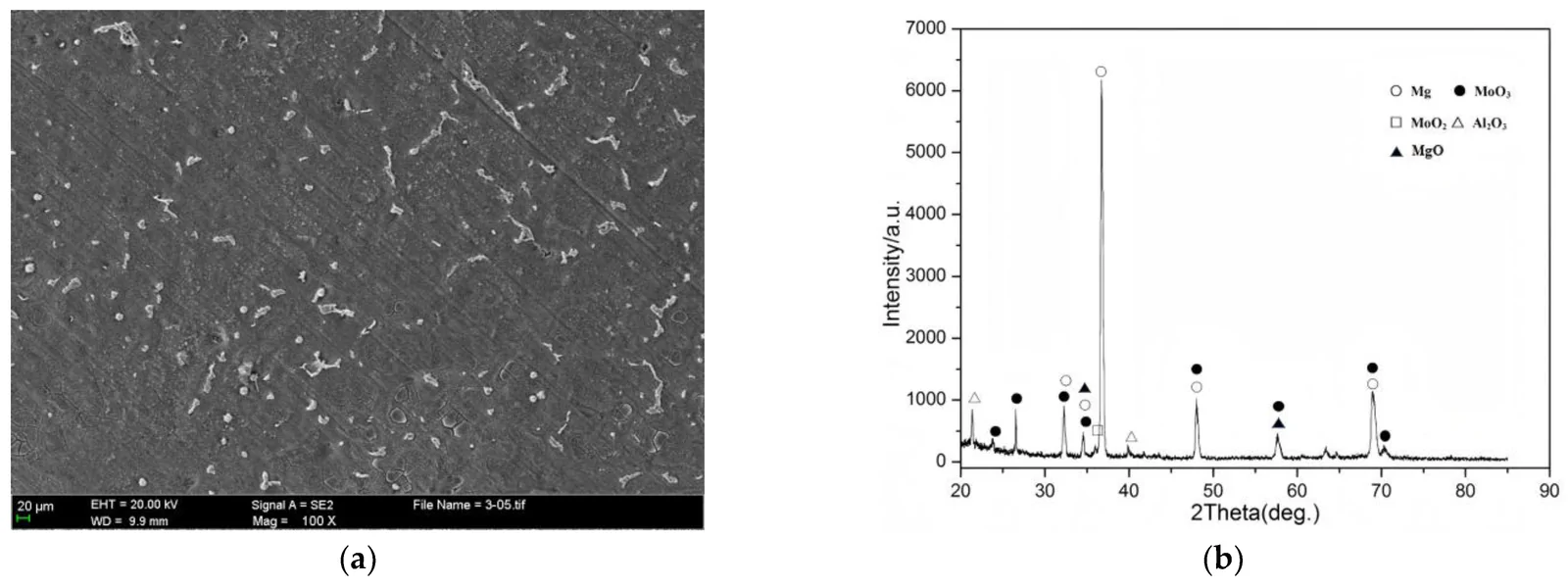
Scanning electron microscope and X-ray diffraction of molybdate conversion film of AM60B-MM magnesium alloy. (**a**) Scanning electron microscope of molybdate conversion film of AM60B-MM magnesium alloy; (**b**) X-ray diffraction of molybdate conversion film of AM60B-MM magnesium alloy.

Figure 2 displays the surface features of the AM60B-MM alloy subjected to molybdate conversion treatment, with the SEM image in Figure 2a and the XRD pattern in Figure 2b. The results of the observation show that the conversion film is uniform and dense, and the surface has some irregular microcracks. In the film layer, a number of white crystalline substances are present, and the residual metal salt in the film-forming liquid is attached during the drying process. The results of X-ray diffraction analysis of molybdate conversion films of the AM60B-MM magnesium alloy are mainly composed of MgO, MoO_2_, MoO_3_ and Al_2_O_3_. Molybdate is weakly oxidized in the acidic medium and is reduced on the surface of the magnesium alloy [22]:

MoO_4_^2−^ + Mg + 2H^+^ → MoO_2_ + MgO + H_2_O (1)

MoO_4_^2−^ + Mg + 2H^+^ → MoO(OH)_2_ + MgO (2)

MoO_4_^2−^ + 2H^+^ → H_2_MoO_4_ → MoO_3_ + H_2_O (3)

The products of the above reaction together form the conversion film, and the reaction shown in Equation (3) mainly occurs in the surface layer. Molybdate is formed at the interface between molybdate and metal/solution, which is decomposed into MoO_3_ during the drying process at room temperature.

**Figure 3:**
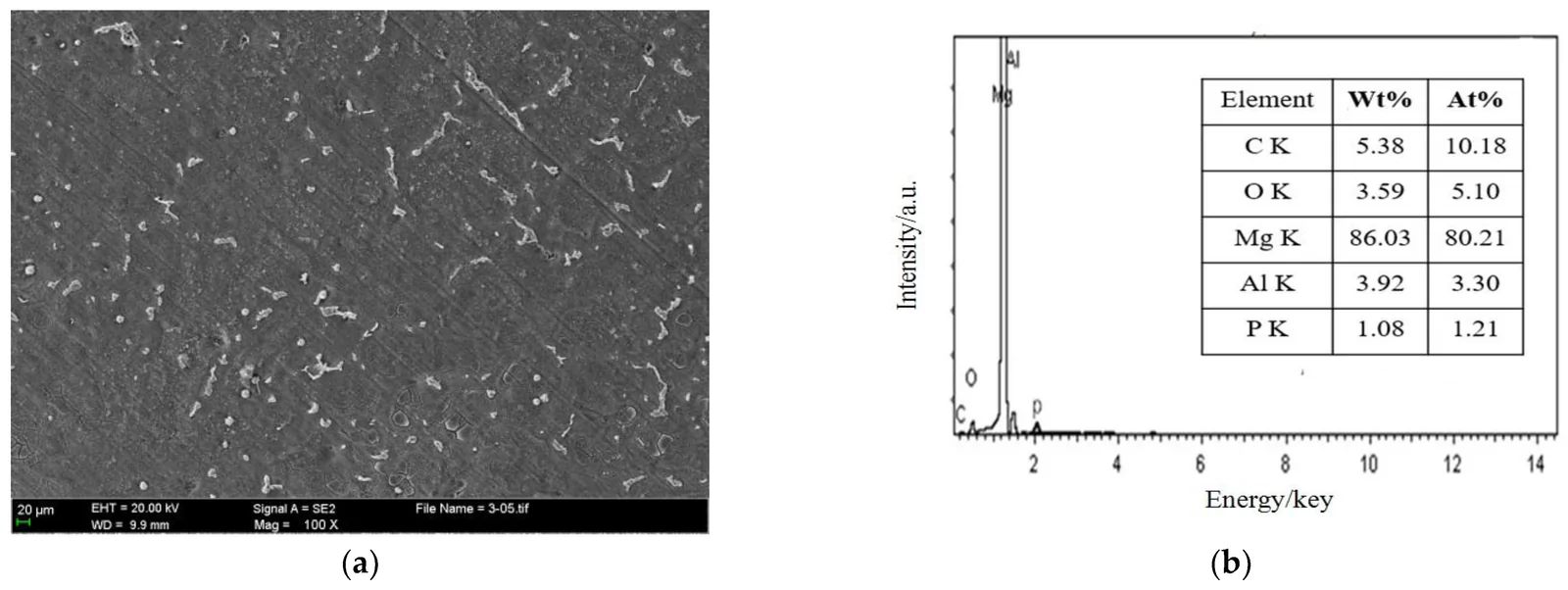
Scanning electron microscope and energy spectrum analysis of phytic acid conversion film of AM60B-MM magnesium alloy. (**a**) Scanning electron microscope of phytic acid conversion film of AM60B-MM magnesium alloy; (**b**) energy spectrum analysis of phytic acid conversion film of AM60B-MM magnesium alloy.

Figure 3 shows the surface morphology and elemental composition of the phytic acid conversion coating on the AM60B-MM alloy, where Figure 3a provides the SEM micrograph and Figure 3b presents the result of energy spectrum analysis. The phytic acid conversion film exhibits a uniform, dense, smooth surface free of obvious defects. The results of energy spectrum analysis showed that the surface of the phytic acid conversion film of the AM60B-MM magnesium alloy was mainly composed of Mg, O, Al, C and P, with mass fractions of 86.03%, 3.59%, 3.92%, 5.38% and 1.08%, respectively. Specifically, Mg and Al are intrinsic elements from the magnesium alloy substrate, while C, P and O come from phytic acid that reacts with the alloy matrix and adsorbs onto the surface. As a natural polydentate chelating agent [23], phytic acid has high affinity to positively charged metal ions on the magnesium alloy surface and forms stable magnesium phytate and aluminum phytate compounds through chemical adsorption.

### 3.2. Semiconductor Behavior of Three Kinds of Chemical Conversion Films

The oxide film formed on the metal surface usually shows semiconductor properties, and the semiconductor properties of the oxide film also directly affect the corrosion resistance of the metal. When the oxide film is in contact with the electrolyte solution, the space charge capacitance (*C_SC_*) and the potential (*E*) can be analyzed using the Mott–Schottky equation [24].

**Figure 4:**
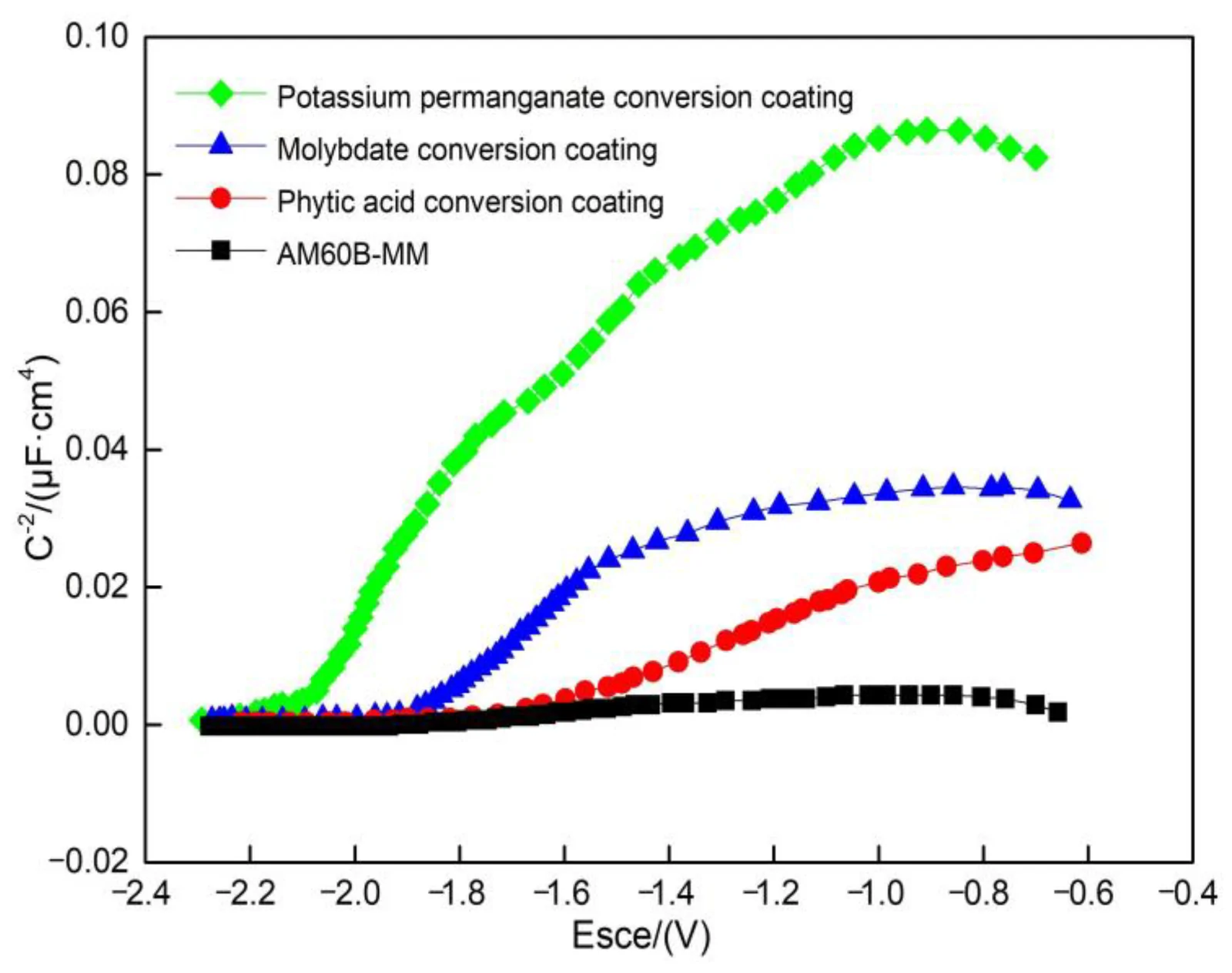
Mott–Schottky curves of AM60B-MM magnesium alloy and three kinds of chemical conversion films in 3.5%NaCl solution.

Figure 4 shows the Mott–Schottky curve of the AM60B-MM magnesium alloy and three chemical conversion films in 3.5% NaCl solution. The Mott–Schottky curve is performed with linear fitting in a potential range of −2.0 to −1.0 V.

For n-type semiconductor, the Mott–Schottky is:

(4) $$\frac{1}{C_{SC}^{2}}=\frac{2}{{\varepsilon \varepsilon }_{0}eN_{D}}\left(E-E_{FB}-\frac{kT}{e}\right)$$

For p-type semiconductor, the Mott–Schottky is:

(5) $$\frac{1}{C_{SC}^{2}}=\frac{2}{{\varepsilon \varepsilon }_{0}eN_{A}}\left(E-E_{FB}-\frac{kT}{e}\right)$$

where, *C_SC_*—space charge layer capacitance; *ε*—dielectric constant of the film layer; *ε*_0_—permittivity of vacuum; *e*—electron charge; *N_D_*, *N_A_*—donor and acceptor density, respectively; *E*—applied potential; *E_FB_*—flat band potential; *k*—Boltzmann constant; *T*—degree kelvin.

The slope of the linear equation can be used to obtain donor density (*N_D_*), while the intercept can be used to determine the flat band potential (*E_FB_*). A positive slope in the linear region indicates n-type semiconductor behavior of the film. Accordingly, the carrier density and flat band potential of each conversion coating were extracted from the corresponding Mott–Schottky plot, and the results are listed in Table 1. In the range of −2.0 to −1.0 V, all three conversion films exhibit well-defined linear Mott–Schottky relationships with positive slopes, characteristic of n-type semiconductors. Notably, the slopes of the linear regions for the conversion films are distinctly larger than that of the bare AM60B-MM magnesium alloy substrate.

The flat band potential *E_FB_* is closely related to the pitting corrosion potential of the AM60B-MM magnesium alloy matrix. When *E_FB_* decreases, the pitting corrosion potential increases [25]. As can be seen from Table 1, the *E_FB_* of the three chemical conversion membranes obtained by fitting is between approximately −2.277 V and −2.028 V, all of which are negatively shifted relative to the value of −1.963 V for the bare alloy substrate. This result indicated that the corrosion resistance of the magnesium alloy was significantly improved after chemical conversion film treatment.

The change in donor density *N_D_* reflects the change in the composition and structure of the chemical conversion film of the magnesium alloy. A lower *N_D_* indicates a lower probability of destruction or pitting erosion of the chemical conversion film. The slope of the Mott–Schottky curve is inversely proportional to *N_D_*: a larger slope corresponds to a smaller donor density. As shown in Table 1, the *N_D_* value of the AM60B-MM magnesium alloy matrix is 5.939 × 10^21^ cm^−3^, while the *N_D_* value of the three chemical conversion films is greatly reduced to 0.397 × 1021 cm^−3^~0.766 × 10^21^ cm^−3^. This reduction implies decreased electrochemical activity of the conversion coatings, suppressed interfacial electron transfer, and a greatly reduced concentration of point defects (including oxygen vacancies, cation interstitials, and cation vacancies) [26]. The decreased defect density improved the order degree of the conversion film and enhanced the corrosion resistance.

### 3.3. Electrochemical Behavior of Three Kinds of Chemical Conversion Membranes

**Figure 5:**
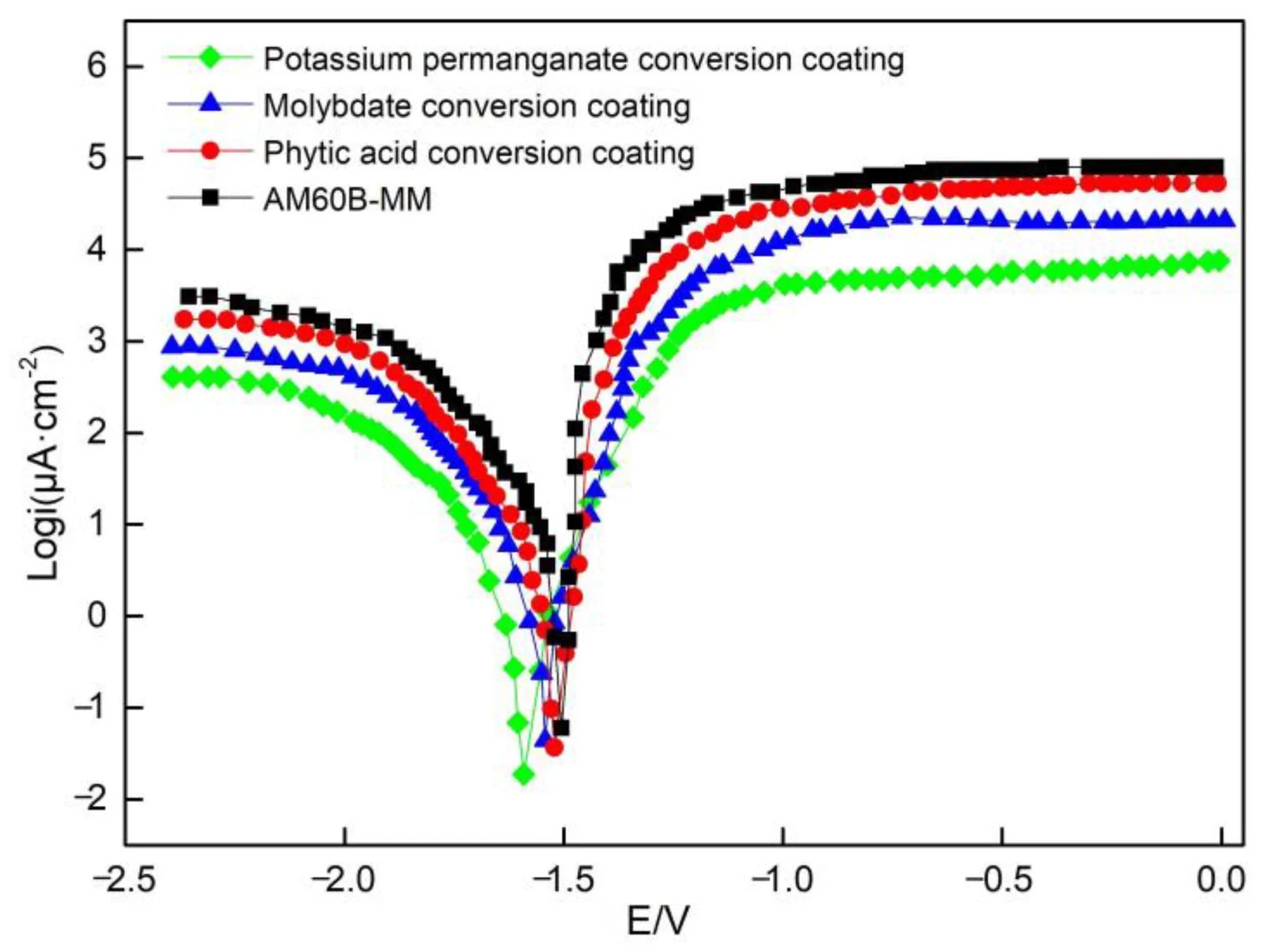
Polarization curves of AM60B-MM magnesium alloy and three kinds of chemical conversion films in 3.5%NaCl solution.

The polarization curves of AM60B-MM and the three chemical conversion films in 3.5% NaCl solution are shown in Figure 5. All samples exhibit a rapid rise in anodic current density with increasing anodic polarization potential, whereas the three conversion films suppress the anodic dissolution kinetics to varying degrees, with their polarization curves shifting toward lower-current-density regions. The corresponding electrochemical parameters extracted via Tafel extrapolation are summarized in Table 2. Compared with the AM60B-MM magnesium alloy, the corrosion potential of the three kinds of conversion film samples moves positively, and the corrosion current density decreases. This shows that the corrosion tendency of the AM60B-MM magnesium alloy is higher than that of the conversion film sample, because the lower the corrosion potential, the earlier the alloy begins to corrode in the corrosion environment, and vice versa. The corrosion rate is determined by the corrosion current density, and the higher the corrosion current density, the faster the corrosion rate [27], so the corrosion rate of the AM60B-MM magnesium alloy is higher than that of the three kinds of conversion film samples. This is due to the formation of three kinds of surface treatment samples. These films hinder the diffusion of anodic dissolution products from the alloy surface and decelerate the overall electrochemical corrosion process.

**Figure 6:**
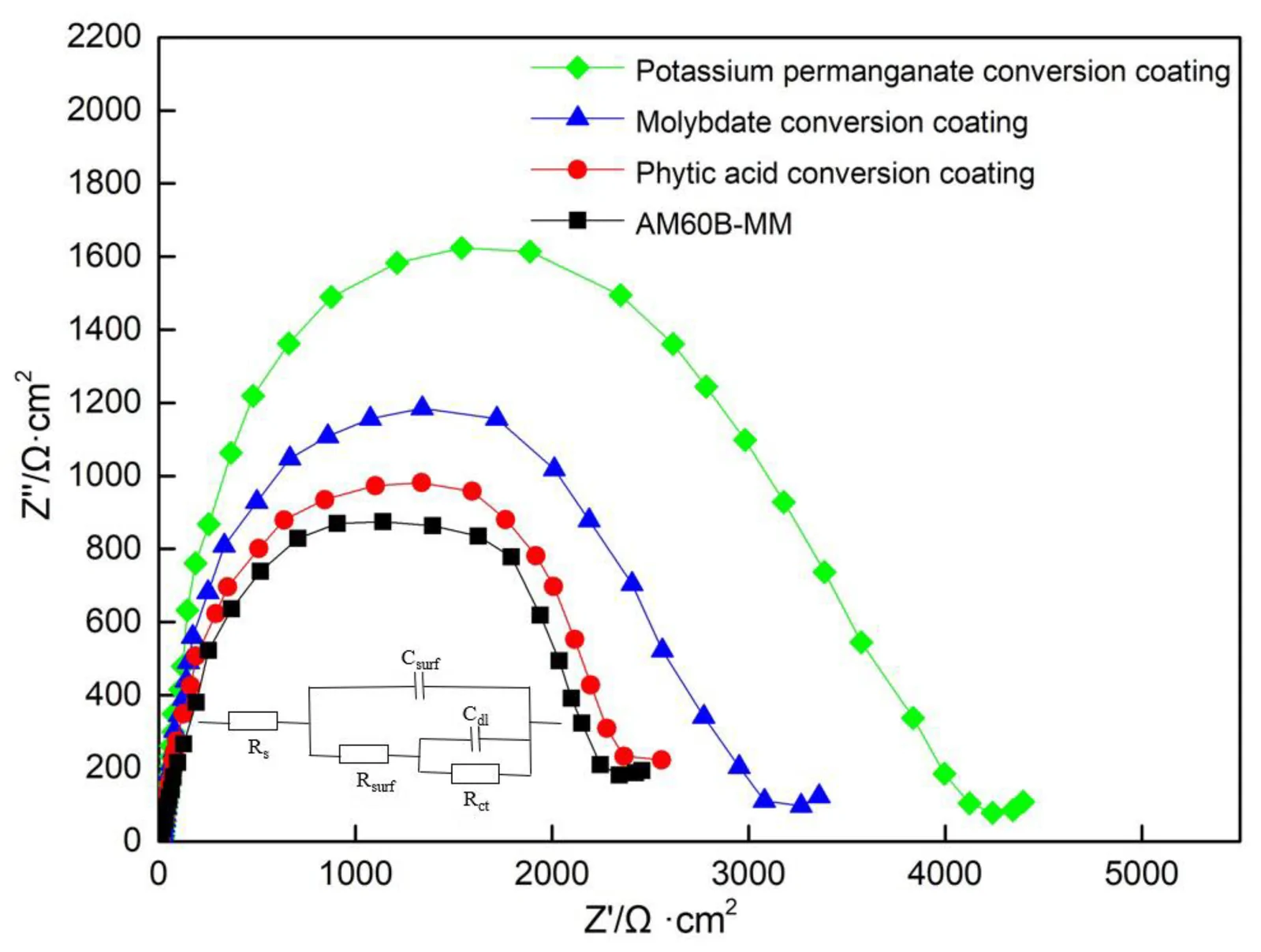
3.5% Nyquist plots of AM60B-MM magnesium alloy and three chemical conversion membranes in NaCl solution.

The Nyquist spectra of the AM60B-MM magnesium alloy and three kinds of chemical conversion films in 3.5%NaCl solution are shown in Figure 6. It can be seen from the diagram that the AM60B-MM magnesium alloy and its three chemical conversion films all show a single capacitive arc, which indicates that the electrode reaction of the four samples is controlled by charge transfer in the range of the test. The arc diameter of the magnesium alloy matrix is small before chemical conversion but increases to varying degrees after three kinds of chemical transformation treatment. It is generally believed that the diameter of the capacitive loop directly reflects the magnitude of charge transfer resistance at the corrosion interface. A larger loop diameter corresponds to higher interfacial impedance and lower double-layer capacitance, which is a well-recognized indicator of enhanced corrosion resistance [28]. According to Figure 6, the corrosion resistance of the permanganate conversion film in 3.5%NaCl solution is better than that of the molybdate conversion film and phytic acid conversion film, and much better than that of the AM60B-MM magnesium alloy, which is consistent with the polarization curve in Figure 5.

An equivalent circuit (illustrated in Figure 6) [29] is used to fit these data, and the results are shown in Table 3. Among them, R_s_ is solution resistance, R_surf_ and C_surf_ are film resistance and film capacitance produced by conversion film, R_ct_ is the charge transfer resistance of the corrosion reaction, C_dl_ is the double-layer capacitance between the sample and solution. The charge transfer resistance R_ct_ of the three conversion films increases significantly, which indicates that the greater the resistance between the electrode and the electrolyte solution, the stronger the corrosion resistance of the samples. At the same time, the double-layer capacitance C_dl_ is related to the porosity of the oxide film on the surface of the magnesium alloy. The C_dl_ values of the three chemical conversion films are lower than those of the AM60B-MM magnesium alloy, which indicates that the conversion film formed on the surface of the magnesium alloy is compact, plays the role of sealing pores, reduces the porosity, and can prevent the erosive Cl^−^ ion from entering the surface of the alloy, thus effectively protecting the matrix alloy. In addition, the film resistance R_surf_ value of the three kinds of chemical conversion films increases, and the membrane capacitance C_surf_ value decreases, showing the same trend as R_ct_ and C_dl_.

### 3.4. Friction and Wear Behavior of Three Kinds of Chemical Conversion Films

**Figure 7:**
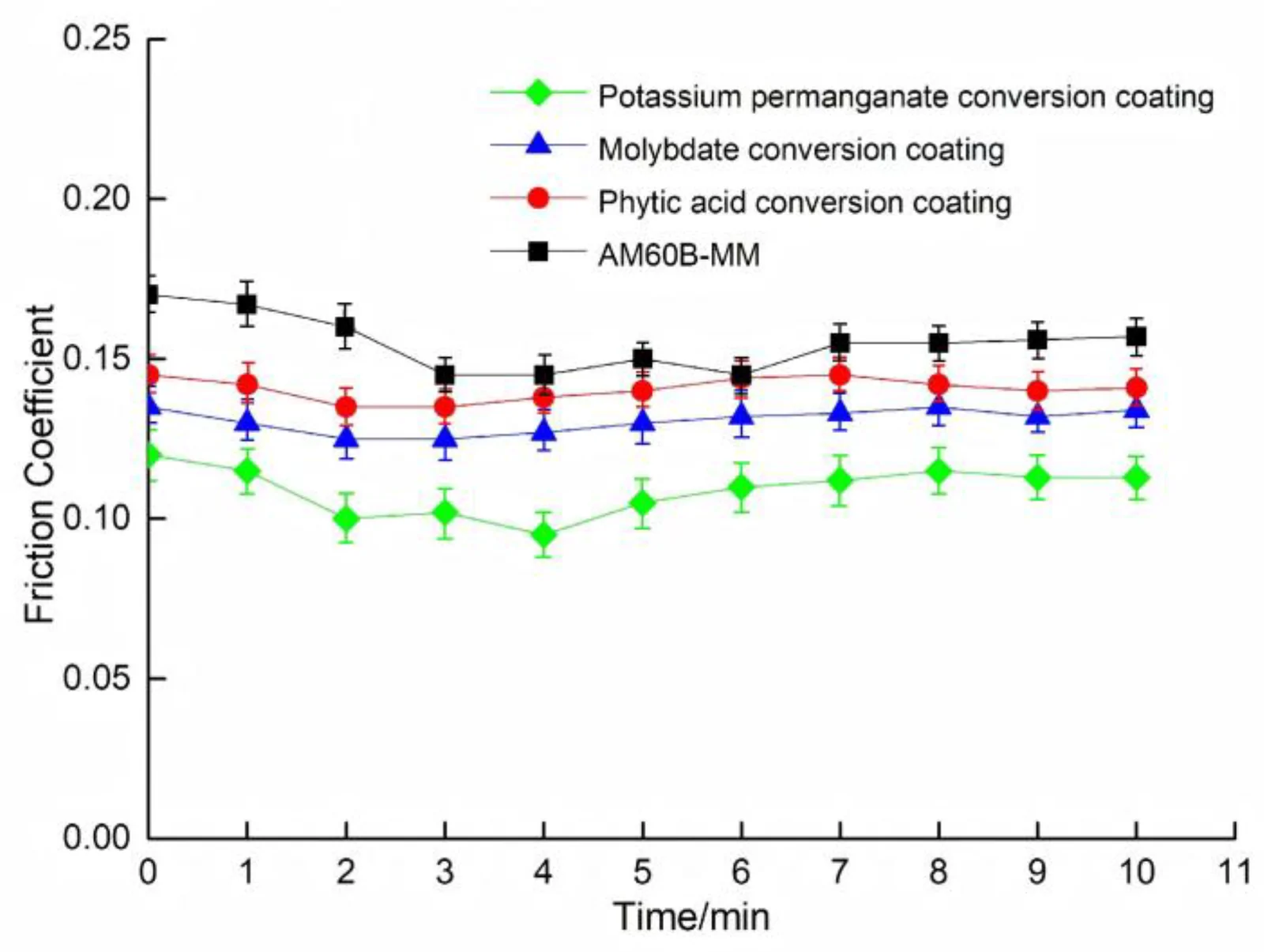
Relationship curve of friction coefficient of AM60-MM magnesium alloy and three kinds of chemical transformed films in 3.5%NaCl solution with time.

Figure 7 shows the variation in the friction coefficient of the AM60-MM alloy and three kinds of chemical transformed films with time in 3.5%NaCl solution. The bare AM60B-MM alloy presents a relatively high friction coefficient of approximately 0.157. In contrast, the permanganate, molybdate, and phytic acid conversion coatings all yield markedly lower friction coefficients, measured at 0.113, 0.134, and 0.141, respectively, corresponding to improved wear resistance. The specific wear rate of the AM60-MM alloy is 2.1342 mm^3^·N^−1^·m^−1^, while those of the permanganate, molybdate and phytic acid conversion film-coated AM60-MM are 0.7138, 1.0221 and 1.3789 mm^3^·N^−1^·m^−1^, respectively. The wear rates were all reduced after film formation.

**Figure 8:**
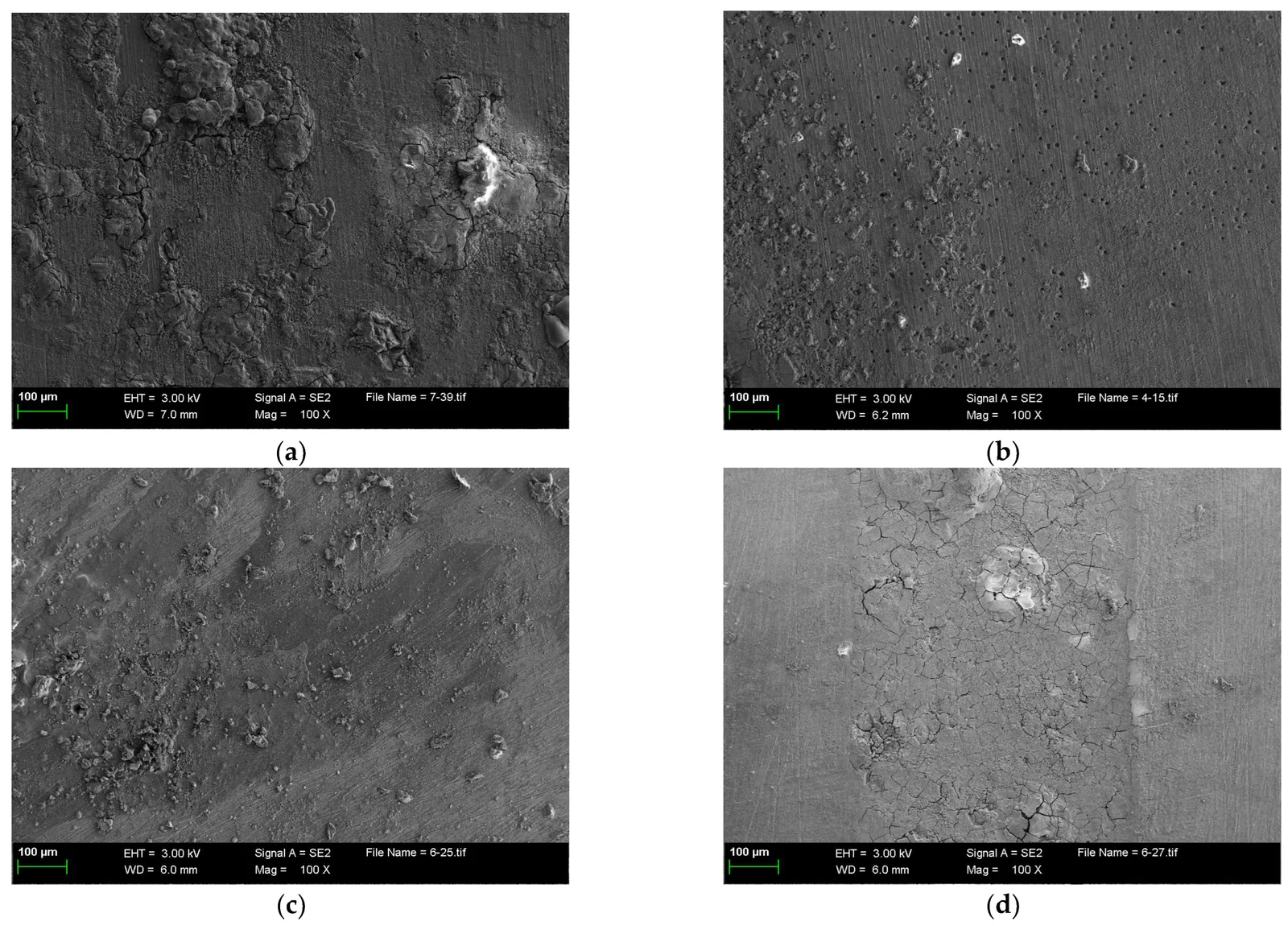
Surface corrosion morphology of AM60-MM magnesium alloy and three kinds of chemical transformed films in 3.5%NaCl solution after friction. (**a**) AM60-MM magnesium alloy, (**b**) permanganate conversion film of AM60B-MM magnesium alloy, (**c**) molybdate conversion film of AM60B-MM magnesium alloy, (**d**) phytic acid conversion film of AM60B-MM magnesium alloy.

Figure 8 presents the surface morphologies of the bare AM60B-MM alloy and the three conversion films after the friction–wear test. For the bare AM60B-MM substrate, severe wear damage is observed after sliding, with distinct wear tracks formed on the friction surface that exhibit typical abrasive wear characteristics. A large area of flake debris appears along the wear mark, the wear debris size is large, and the wear surface is accompanied by crack formation. As the crack continues to expand, the flake debris falls off (Figure 8a). However, the surface wear of the three kinds of chemical conversion films is mild, and there are small granular debris and fine microcracks on the surface of the conversion films; the wear debris size is reduced (Figure 8b–d), and the wear resistance is improved.

Wear debris generated from both the bare AM60-MM alloy and the three conversion-coated specimens appears as fine black powder. Energy-dispersive spectroscopy (EDS) characterization identifies the debris as a mixture of magnesium oxides and metallic magnesium wear fragments. The results show that the content of O element (wt%) of the permanganate, salt and phytic acid conversion film is 25.69%, 23.33% and 18.72%, respectively, which is much higher than that of the corresponding AM60-MM magnesium alloy matrix (12.51 wt%). This result indicates that the surface of the magnesium alloy has been oxidized and worn. The conversion films improve wear resistance via two synergistic mechanisms. First, the as-formed surface layers serve as a solid lubricant during sliding while also hindering the initiation and propagation of surface microcracks. This reduces wear debris size, weakens the abrasive cutting effect of hard particles, and suppresses delamination of the surface layer during friction. Second, magnesium oxide phases within the conversion coatings exhibit higher hardness than the magnesium matrix [30], acting as a hard load-bearing component on the contact surface. This effect alleviates direct metal-to-metal contact and plastic deformation of the soft substrate, further enhancing the overall wear resistance of the alloy.

The corrosion resistance and wear resistance of the sample in the 3.5% NaCl solution are determined according to the flat charging position, the corrosion current density, the tolerance arc radius and the friction coefficient. The wear resistance of the sample is as follows: the permanganate conversion film, the acid conversion film, the phytic acid conversion film and the AM60B-MM. The corrosion resistance and wear resistance of the permanganate conversion film are the best. Because of the large amount of MnO_4_^−^ in the potassium permanganate conversion liquid, oxides of MnO, MnO_2_, Mn_2_O_3_, Mn_3_O_4_ and the like with good chemical stability are generated and deposited on the surface of the magnesium alloy to form a chemical conversion film, covering the substrate completely. This reduces the donor density, enhances the anode polarization, improves the charge transfer resistance, and inhibits the Mg dissolving-type anode reaction process: Mg → Mg^2+^ + 2e. The permanganate conversion film is a porous structure, with voids and cracks in the film layer. After the hole-sealing treatment, the defect of the film layer is filled, making the surface of the conversion film more compact and uniform, with fewer defects (Figure 1a), and the chemical–electrochemical stability between the interfaces is improved [31]. This can effectively prevent the penetration of the corrosion medium, enhance the isolation effect of the conversion film on the corrosive environment medium, and improve the corrosion resistance and the wear resistance.

## 4. Conclusions

1. Mott–Schottky measurements in 3.5%NaCl solution show that all three as-fabricated treatments present liner Mott–Schottky plots with positive slopes, exhibiting n-type semiconductor characteristics. The reduced donor density (*N_D_*) and negatively shifted flat band potential (*E_FB_*) indicate that chemical conversion treatment effectively improves the corrosion resistance of the La-Ce mischmetal AM60B magnesium alloy.
2. Short-term electrochemical tests confirm that the three conversion films decrease the corrosion current density, expand the capacitive impedance arc radius, increase the charge transfer resistance, and reduce the double-layer capacitance. These parametric changes collectively contribute to the improved electrochemical corrosion performance of the magnesium alloy substrate.
3. Compared with the untreated AM60B alloy, all three chemical conversion films show significantly lower friction coefficients and reduced specific wear rates, demonstrating greatly improved tribological properties. The wear mechanism of the conversion film surfaces in 3.5 wt% NaCl solution is dominated by abrasive wear, accompanied by moderate oxidative wear.
4. Among the three conversion films, the permanganate-based coating achieves the optimal comprehensive performance with the best corrosion and wear resistance in 3.5 wt% NaCl solution. This superiority originates from its more uniform and compact film microstructure, which establishes a more robust physical barrier to suppress the infiltration of corrosive media and strengthen the isolation of the substrate from the corrosive environment.

## Figures and Tables

**Table 1:** M-S fitting data for AM60B-MM magnesium alloy and three chemical conversion films.

Kind	*N_D_*/cm^−3^	*E_FB_*/V
AM60B-MM	5.939 × 10^21^	−1.963
Permanganate conversion membrane	0.397 × 10^21^	−2.277
Metaconic acid conversion membrane	0.570 × 10^21^	−2.284
Phytic acid conversion membrane	0.766 × 10^21^	−2.028

**Table 2:** Results of dynamic potential polarization curve fitting.

Alloy	E_corr_/V	i_corr_/μA·cm^−2^	Corrosion Rate/mm·a^−1^
AM60B-MM	−1.465	30.142	0.664
Phytic acid conversion membrane	−1.461	24.527	0.541
Molybdate conversion film	−1.452	21.235	0.468
Potassium permanganate conversion membrane	−1.441	16.568	0.365

**Table 3:** Impedance spectrum data fitting results.

Alloy	R_s_ Ω·cm^2^	C_surf_ μF·cm^−2^	R_surf_ Ω·cm^2^	C_d_ μF·cm^−2^	R_ct_ Ω·cm^−2^
AM60B-MM	42.41	0.981 × 10^−5^	1750	3.126 × 10^−5^	1524
Potassium permanganate conversion membrane	39.32	0.263 × 10^−5^	2529	0.325 × 10^−5^	3251
Molybdate conversion film	40.34	0.569 × 10^−5^	2163	1.124 × 10^−5^	2678
Phytic acid conversion membrane	38.93	0.683 × 10^−5^	1981	1.921 × 10^−5^	2678

## Data Availability

The original contributions presented in this study are included in the article. Further inquiries can be directed to the corresponding author.

## References

[1] Elajjani A., Feng Y., Ni W., Xu S., Sun C., Feng S. (2025). Investigation of Thermal Deformation Behavior in Boron Nitride-Reinforced Magnesium Alloy Using Constitutive and Machine Learning Models. Nanomaterials.

[2] Fattah-Alhosseini A., Fardosi A., Karbasi M., Kaseem M. (2024). Advancements in enhancing corrosion protection of Mg alloys: A comprehensive review on the synergistic effects of combining inhibitors with PEO coating. J. Magnes. Alloys.

[3] Maqsood M.F., Raza M.A., Rehman Z.U., Tayyeb A., Makhdoom M.A., Ghafoor F., Latif U., Khan M.F. (2022). Role of Solvent Used in Development of Graphene Oxide Coating on AZ31B Magnesium Alloy: Corrosion Behavior and Biocompatibility Analysis. Nanomaterials.

[4] Eppensteiner F.W., Jenkin M.R. (1999). Chromate conversion coatings. Met. Finish..

[5] Wu L., Dong J., Zheng X., Wang Z., Sun X., Shang X., Ke W., Wang C. (2025). Effect of K_2_HPO_4_ Concentration on the Formation, Structure, Composition and Protectiveness of Conversion Coating Deposited on AZ31 Magnesium Alloy. Acta Metall. Sin.-Engl. Lett..

[6] Hsiao T.H., Lin C.-S. (2024). Mn oxide conversion coating on AZ31B Mg alloy with hexafluorozirconate as an inhibitor in the permanganate conversion bath. Appl. Surf. Sci..

[7] Zhao M., Wu S., Luo J., Fukuda Y., Nakae H. (2006). A chromium-free conversion coating of magnesium alloy by a phosphate–permanganate solution. Surf. Coat. Technol..

[8] Kharitonov D.S., Zimowska M., Ryl J., Zieliński A., Osipenko M.A., Adamiec J., Wrzesińska A., Claesson P.M., Kurilo I.I. (2021). Aqueous molybdate provides effective corrosion inhibition of WE43 magnesium alloy in sodium chloride solutions. Corros. Sci..

[9] Xu R., Li Y., Fan B., Weng Y., Zhou Y., Yan F. (2023). One-step preparation of molybdate-stannate-tungstate composite conversion coating on magnesium alloy AZ91D and its microstructure and corrosion resistance. Int. J. Electrochem. Sci..

[10] Xu Y., Guo Y., Li G., Lian J. (2022). Biodegradable phytic acid conversion coatings on magnesium alloy for temporary orthopedic implant: A review. Prog. Org. Coat..

[11] Bian D., Chu X., Xiao J., Tong Z., Huang H., Jia Q., Liu J., Li W., Yu H., He Y. (2023). Design of single-phased magnesium alloys with typically high solubility rare earth elements for biomedical applications: Concept and proof. Bioact. Mater..

[12] Liang C., Wang S., Huang B., Zhihong Z., Shuchun Z., Jing R. (2015). Effects of Lanthanum and Cerium Mixed Rare Earth Metal on Abrasion and Corrosion Resistance of AM60 Magnesium Alloy. Rare Met. Mater. Eng..

[13] Ijiri M., Kato F., Yoshimura T., Nakatsugawa I., Chino Y., Kikuchi S. (2025). Effects of multifunction cavitation treatment during chemical conversion coating on compounds formed on AZ31 magnesium alloy surface and their electrochemical characteristics. Surf. Coat. Technol..

[14] Amini R., Kardar P. (2025). Rare earth-based conversion coatings under scrutiny: A comprehensive review. J. Rare Earths.

[15] Liu W., Cao F., Chang L., Zhang Z., Zhang J. (2009). Effect of rare earth element Ce and La on corrosion behavior of AM60 magnesium alloy. Corros. Sci..

[16] Zhao J., Lv R., Yang X., Yan D., Dai T., Wang J., Xu G. (2026). Numerical Simulation Study on Corrosion Resistance of Nano-SiC/Micro-arc Oxidized Composite Coating on Magnesium Alloy Surface. JOM.

[17] Safari A., Mozammel M., Emarati S.M., Khalil-Allafi J., Hosseini M. (2024). Impact of voltage variation on topography, hydrophobicity, and corrosion behavior of the CeO_2_-TiO_2_ nanocomposite coating on AM60B Mg alloy prepared by plasma electrolyte oxidation. Surf. Topogr. Metrol. Prop..

[18] Hidalgo Viteri J., Cotolan N., Lupan A., Brânzanic A.M.V., Turdean G.L. (2024). A poly(methyl methacrylate)–ibuprofen composite film as anticorrosive coating of Ti–6Al–4V surface. J. Solid State Electrochem..

[19] Yan T.T., Chen Q.H., Wang Y.Z., Jiang Z. (2014). Effect of permanganate conversion treatment on in vitro corrosion resistance of AZ31B magnesium alloy. J. Mater. Prot..

[20] Yang L., Li J., Yu X., Zhang M. (2008). Molybdate conversion coatings on AZ31 magnesium alloy. Chin. J. Nonferrous Met..

[21] Zhang Z. (2015). Impacts of the phytic acid conversion film on electrochemical corrosion of AZ31 magnesium alloy. Light Alloy Fabr. Technol..

[22] Zhu H., Li X., Guan X., Shao Z. (2021). Effect of Molybdate Conversion Coating of Magnesium Alloy Reinforced by Micro-arc Oxidation. Met. Mater. Int..

[23] Fu C., Zhang J., Guo Y., Hu J., Li C., Hu Y., He S., He Y., Bai Y. (2025). Phytic acid-modified Ce-MOF for enhancing the anticorrosive performance and self-healing properties of waterborne epoxy coating. Prog. Org. Coat..

[24] Li J., Liang C., Huang N. (2014). Effect of B-Mo-W Complex Inhibitor on Corrosion of Mild Steel in 55% LiBr Solution. J. Mater. Eng. Perform..

[25] Ningshen S., Kamachi Mudali U., Mittal V.K., Khatak H.S. (2007). Semiconducting and passive film properties of nitrogen-containing type 316LN stainless steels. Corros. Sci..

[26] Liu Y.P., Song W.H., Chen C.G., Yin L., Guo C.Z. (2013). Corrosion mechanism of anodic oxidation film on AZ31 magnesium alloy in sodium chloride solution. J. Mater. Prot..

[27] Zhou M., Liu C.M., Gao Y.G., Xu S.Y., Jiang S.N. (2019). Corrosion behavior of Cu-containing AZ31 magnesium alloy. Chin. J. Nonferrous Met..

[28] Li H.M., Xie G., Li R.X., Yu X.H., Hou Y.Q. (2018). Comparison of corrosion resistance and discharge behavior of rare earth magnesium alloy, AZ31 and AZ91 negative electrode materials. Hot Work. Technol..

[29] Correa P.S., Malfatti C.F., Azambuja D.S. (2011). Corrosion behavior study of AZ91 magnesium alloy coated with methyltriethoxysilane doped with cerium ions. Prog. Org. Coat..

[30] Huang W.J., Hou B., Pang Y.X., Zhang J., Zhou Z.R. (2005). Fretting wear behavior of two kinds of die cast magnesium alloy. Tribology.

[31] Lee Y.L., Chu Y.R., Li W.C., Lin C.S. (2013). Effect of permanganate concentration on the formation and properties of phosphate/permanganate conversion coating on AZ31 magnesium alloy. Corros. Sci..

